# Fasting-Evoked En Route Hypoglycemia in Diabetes (FEEHD): An Overlooked Form of Hypoglycemia in Clinical Practice

**DOI:** 10.1155/2018/1528437

**Published:** 2018-10-24

**Authors:** Saleh Aldasouqi, Samia Mora, Gaurav Bhalla, Naveen Kakumanu, William Corser, George Abela, Mohammad Dlewati, Kathleen Estrada, Abdul Almounajed, Tarek Tabbaa, Jamal Hammoud, Cathy Newkirk

**Affiliations:** ^1^Division of Endocrinology, Department of Medicine, College of Human Medicine, Michigan State University, USA; ^2^Division of Preventive Medicine, Department of Medicine, Brigham and Women's Hospital, Harvard Medical School, USA; ^3^College of Osteopathic Medicine, Michigan State University, USA; ^4^Division of Cardiology, Department of Medicine, College of Human Medicine, Michigan State University, USA; ^5^University of Michigan-Flint, USA; ^6^College of Human Medicine, Michigan State University, USA; ^7^Wayne State University, USA; ^8^College of Human Medicine-Flint, Michigan State University, USA; ^9^Michigan State University Extension-Flint, USA

## Abstract

**Objective:**

Many patients with diabetes opt to fast for lab tests, especially for lipid profiles, thus missing breakfast. In parallel, recent studies and international guidelines have indicated that routine fasting for lipid panels may not be necessary. Missing breakfast while fasting for lab tests may invoke hypoglycemia, if patients are not properly instructed about adjusting diabetes medications on the night before or on the day of the lab test. Our group described this form of hypoglycemia and introduced the term FEEHD to refer to it (fasting-evoked en route hypoglycemia in diabetes). In a recently published small study, we reported a rate of occurrence of FEEHD of 27.1%. The objective of this study was to evaluate the rate of occurrence of FEEHD in another clinic.

**Methods:**

Patients with diabetes were asked to complete a simple, 2-page survey inquiring about hypoglycemic events while fasting for labs in the preceding 12 months.

**Results:**

A total of 525 patients completed the surveys out of 572 patients invited (91.8% response rate). A total of 363 patients with complete data were analyzed, with a mean age of 60.6 (SD 12.5) years. A total of 62 (17.1%) patients reported having experienced one or more FEEHD events in the prior 12 months. Of the 269 patients who were at higher risk of FEEHD (on insulin secretagogues or on insulin), 59 (21.9%) reported having experienced FEEHD. Only 33 of FEEHD patients (53%) recalled having contacted their provider regarding the events and only 22 (35%) indicated having received some sort of FEEHD prevention instructions.

**Conclusion:**

Our study shows a significant rate of occurrence of FEEHD in the real world (a clinical practice). FEEHD is especially dangerous, as patients often commute (drive) to and from the laboratory facility (potential risk of traffic accidents). Given study limitations, further studies are needed to assess prevalence of FEEHD in other settings and in the general populations.

## 1. Introduction

Hypoglycemia in patients with diabetes, defined as blood glucose below 70 mg/dl [1], is one of the most common adverse events in patients taking insulin or oral hypoglycemic agents. Therefore, hypoglycemia is practically the rate limiting step in aggressive control of diabetes [1]. Hypoglycemic events are unpleasant and are associated with negative emotional, social, and behavioral consequences for patients and in extreme cases leading to life-threatening arrhythmias and sudden cardiac death [2, 3]. Fear of hypoglycemia is also an important contributor to worse quality of life for patients with diabetes, as such episodes can lead to patient dissatisfaction with management and also affect physicians' aggressiveness in optimal management of diabetes leading to underutilization of treatment regimens and failure to achieve optimal glycemic targets [4, 5].

A conceivably overlooked cause of hypoglycemia is the procurement of fasting labs, of which the lipid panel is the most commonly ordered fasting lab test. Traditionally, guidelines have recommended routine fasting lipid panels [6, 7]. Recently, though, the notion of routine fasting lipid panel in every patient has been challenged, with recent European and Canadian guidelines endorsing nonfasting lipid testing for routine clinical care and management decisions [8–11].

By ordering fasting labs, we may not only be inconveniencing patients, but we may also be inadvertently putting them at risk for hypoglycemia [12–14]. A case report from Thailand [15] reported on a patient who had hypoglycemia in the waiting room of a laboratory, while waiting for a fasting lab. The patient had a sudden cardiac arrest that led to the death of the patient (blood glucose during resuscitation was later reported to be 0 mg/dl). The patient was on a sulphonylurea for her diabetes [15].

The acronym FEEHD was proposed to refer to this form of hypoglycemia, and the acronym stands for “fasting-evoked en route hypoglycemia in diabetes” [11, 13, 14, 16, 17]. FEEHD is arbitrarily defined by the following criteria: a hypoglycemic event (blood glucose below 70 mg/dl) in patients with diabetes who take insulin or sulfonylurea, or both, who fast overnight for lab tests, and who commute to the laboratory facility while fasting. Most of these hypoglycemia episodes go unreported and the patients are rarely given instructions to change or adjust their medications prior to fasting.

A recent pilot nonrandomized study [16] demonstrated a significant occurrence of FEEHD episodes in clinical practice: 27.1% of 168 enrolled patients reported having experienced one or more FEEHD events. This prompted the current larger study, with the objective of estimating the rate of occurrence of FEEHD in a different clinical setting (a different clinical practice), aiming at recruiting a higher number of patients.

## 2. Methods

This was a nonrandomized, prospective survey (questionnaire) study. Study participants were patients who were attending the study clinics for initial or follow-up visits. They were enrolled in person by study coinvestigators (undergraduate or medical students). Patient enrollment occurred per the convenience and availability of study coinvestigators (students) to attend clinics, to enroll study patients.

### 2.1. Participants

Adults with diabetes were recruited for this study, which was conducted through a survey instrument at two study sites: two locations of an endocrinology practice. The research protocol was approved by the Institutional Review Board, and all participants gave written informed consent. This study was based on a recently published study, utilizing similar inclusion and exclusion criteria [16]. Briefly, inclusion criteria included adult patients who were able to understand and answer the survey questions, who had a confirmed diabetes diagnosis, and who were taking insulin and/or oral hypoglycemic agents or noninsulin injectable medications. Exclusion criteria included patients who were unable to understand or complete the survey questionnaire even with assistance, patients who were not taking medications regularly, and surveys that were not completely filled.

The study was conducted from March 1 to September 30, 2016, during outpatient clinic appointments at the research sites.

### 2.2. Study Procedures: The Survey Questionnaire

The survey was a simple language 2-page document asking about the duration and type of diabetes, medication use, and any episodes of FEEHD in the preceding 12 months and if any instructions were given to the patient about medication dose adjustment prior to having laboratory tests. The survey was adapted from the questionnaire used in a recent study [16]. At the end of the survey, a template notification was made to instruct patients to notify their care providers of any hypoglycemic events to implement preventive measures.

### 2.3. Statistical Analysis

A series of descriptive statistical analyses were completed to examine for missing data patterns and distribution patterns of key study variables. We then conservatively categorized continuous patient characteristic data (e.g., age in years and years of diabetes duration) into equivalent-sized tertile groups. To inform the configuration of subsequent stepwise logistic regression modeling procedures, a series of Pearson product-moment bivariate correlation procedures were completed to examine for suitable discrete model terms entered into subsequent predictive models.

A basic two-tailed stepwise binary logistic regression predictive modeling procedure was then completed to examine for statistically significant influences of whether fasting lab patients experienced one or more FEEHD episode during the 12-month reporting period. In such a procedure, each model term is introduced one at a time, with model terms showing significance levels of greater than 0.10 removed from the final predictive model. A *p* value level of 0.05 was observed to indicate statistical significance. All analyses were completed using the S.P.S.S. version 24 analytic software.

## 3. Results

A total of 572 patients were invited to take part in the survey (Table 1). A total of 525 patients agreed to complete the surveys. A total of 47 patients either declined or were unable to complete the surveys (response rate, 91.8%). The study coinvestigators listed the causes behind the declination or inability of the 47 patients to complete the survey. These causes were understandable, such as patient citing: “being in a hurry”; “not interested in the study”; or “unable to complete survey”, or the students would not enroll patients due to an ongoing acute illness during the visit such as having a hypoglycemic event.

After the exclusion of 127 (24.2%) patients who reported no fasting labs during the 12 presurvey months and the exclusion of 35 (7%) study participants with missing data, a final analytic sample of 363 respondents (169 men and 194 women) was examined. The detailed statistical analysis was performed on the total number of these patients (i.e., 363 respondents). The mean age of the analytic sample was 60.6 years (SD 12.5), and the duration of their diabetes averaged 16.0 years (SD 11.5). A total of 298 (81.6%) patients reported a diagnosis of type 2 diabetes mellitus. Of the total 363 patients included in the analysis, 131 (36%) reported that they were previously educated about how to fast for labs and how to take preventive measures such as medication adjustment (e.g., “reduce your insulin dose,” “have someone drive you to get your lab drawn”).

A total of 62 out of 363 patients (17.1%) reported having experienced at least one FEEHD event in the preceding 12 months. Of these 62 patients who reported FEEHD events, 7 patients (11%) reported multiple FEEHD events. Only 33 of all the 62 FEEHD patients (53%) recalled having contacted their provider regarding the events. Only 22 of these 62 patients (35%) indicated having received some sort of FEEHD prevention instructions, following notification of provider.

Regarding their medication regimens, the number of patients on hypoglycemia-inducing oral hypoglycemic agents (OHAs), namely, sulfonylureas and meglitinide analogues, insulin, or both, was 36, 215, and 18, respectively. Thus, the total at-risk population was 269 out of 363 patients (74.1%). Of the “At-risk” patients, 59 out of 269 patients (21.9%) had one or more episodes of FEEHD during the preceding 12 months. A total of 35 out of these 59 patients (59%) could recall their specific blood glucose readings at the time. Their blood glucose readings averaged 56 mg/dl (SD 10), with a range from 32 to 65 mg/dl.

Of the total 363 patients analyzed, 149 patients (41%) reported hypoglycemic events related to any cause (including those reporting FEEHD events) in the preceding 12 months. These 149 patients who reported “all-cause” hypoglycemic events could specifically recall at least one specific circumstance related to their hypoglycemia episode(s). These included (frequently overlapping) reasons such as (a) fasting/eating less during prior night (*n* = 75), (b) exercising (*n* = 55), (c) recent medication changes (*n* = 17), and (d) multiple cited reasons (*n* = 29).

Upon further statistical analysis of the data, major nonsignificant correlations with the occurrence of one or more FEEHD episode(s) included (a) gender (*p* = 0.752), (b) age category (*p* = 0.909), and (c) type of diabetes (*p* = 0.863). However, patient characteristics that were significantly correlated with one or more FEEHD episode(s) included the following:

1. Hypoglycemic symptoms (*p* < 0.001)
2. Frequency of (all-cause) hypoglycemic episodes during past year (*p* < 0.001)
3. Taking insulin (*p* = 0.014)
4. Being on an insulin pump (*p* < 0.001)

## 4. Discussion

These results indicate a high prevalence of FEEHD events (17.1%) in clinical practice, which is relatively consistent with the 27.1% prevalence rate reported in a recently published study [16]. The prevalence in the current study is 21.9% in at-risk patients (e.g., those on hypoglycemia-causing medications such as insulin and/or sulfonylureas). With recent studies [18] indicating increasing use of insulins, it is conceivable that FEEHD may lead to increasing rates of hypoglycemia.

The first study reporting on the occurrence of FEEHD in clinical practice was published as a pilot study in 2011 [12]. The trigger for that study was the clinic's nurses becoming concerned about the repeated calls from the laboratory about low glucose results (sometimes critically low). Those lab results would be available to the lab staff hours after the blood draw earlier in the morning. This study was followed by two studies and a case series [13, 16, 17], in which the occurrence of FEEHD was observed, confirming findings of the first pilot study.

However, the aforementioned studies were limited by sample size and lack of generalizability. In the first study [12], the investigators retrospectively tracked hypoglycemic events from their hospital's laboratory records over a preceding 21-month period. The same investigators then implemented a hypoglycemia prevention program and then undertook a follow-up study [13], following the same protocol of the first study tracking laboratory results in the subsequent 21 months. The investigators observed significant prevention of hypoglycemia (FEEHD). In the 3^rd^ publication (a case series study), 4 cases of FEEHD [17] were captured and were meticulously analyzed, for the purpose of better understanding of the circumstances and causes of the hypoglycemic events. As such, the preceding 3 studies/case series could not address the prevalence of FEEHD. The first study to address prevalence [16] was a small-sized pilot study (*n* = 168), which showed a significant prevalence of FEEHD (27.1%) in a different clinical setting.

Collectively, these studies/case series [12, 13, 16, 17] could not attract significant attention by the mainstream medical communities or health organizations. Therefore, we believe that the results of the current study are more convincing, given the larger sample size. We hope that we have been able to make a stronger case about the actual occurrence of FEEHD, and we believe that it is an overlooked problem. Of concern, our study showed that only 53% of patients reported the FEEHD events to their providers, and only 35% received education regarding prevention of future events, a finding that is consistent with the literature. It has been reported that hypoglycemic events occurring outside of clinics (at home or elsewhere), in general, are often not reported by patients to their health care providers [19, 20].

The rate of occurrence of FEEHD as estimated by our study in the total cohort is lower (17%) than that in the study which was recently published [16] which was (27.1%), though it is still significantly high in the “at-risk” population (21.9%; patients on hypoglycemia-causing medications such insulin and/or sulfonylureas who had fasting labs). This lower rate percentage may be related to different sample sizes, different practice methods, or different patient health education (self-education or education by clinicians).

Of note, our study showed that only 36% of patients received proper instructions about preparing for fasting for labs. We have not found literature addressing if patients with diabetes are educated or informed when fasting labs are ordered, except for sporadic reports [21–23]. A small study by Kackov and associates has found that the majority of outpatients are not well informed about how to fast for lab tests [21]. Only 15% and 19% of patients reported that they were properly informed by a doctor or a nurse, respectively, about preparation for fasting for labs. Furthermore, few other investigators addressed inpatient fasting orders (for various indications) and raised concerns about the appropriateness of these orders [22], as well as the potential risk of hypoglycemia in patients with diabetes [23].

A final note is that hypoglycemia has been linked to increased risk of traffic accidents. As reported by Moghissi, 19% of patients with type 2 diabetes reported that hypoglycemic events often occur during driving [19]. The American Diabetes Association's guidelines [24] state that “Clinically significant hypoglycemia can cause acute harm to the person with diabetes or others, especially if it causes falls, motor vehicle accidents, or other injury.” Given these notions, there is a conceivable risk of traffic accidents due to hypoglycemia of the FEEHD type, if the hypoglycemia is severe. Hence, the utilization of the word “en route” in the acronym, FEEHD, emphasizes the observation that patients drive to and from laboratory facilities [11, 13, 14, 16, 17]. Undoubtedly, it is a routine that patients usually drive themselves to and from lab facilities in the morning for fasting lab tests, typically on the way to work.

In parallel to this ongoing research about the risk of fasting for labs in patients with diabetes (FEEHD), there has been a growing thrust of research questioning the necessity of fasting when ordering lipid profiles, which are the most commonly ordered fasting labs in clinical practice. Emerging deliberations have been raised about the utility of fasting lipid levels with various guidelines in Europe and Canada siding with nonrequirement of fasting lipid panels [8–10, 25, 26]. In the United States, the tradition for fasting labs is deeply entrenched in the psyche of patients and physicians alike. In our clinical practice, it has been noticed that as soon as a patient is informed that labs may be required, one of their first responses is “but, I am not fasting today.”

This deeply rooted belief is not without reason as most US guidelines continue to recommend fasting lipid panels; though on a small scale, some US experts have recently advocated nonfasting lipid panels [25, 26]. These emerging opinions [26] have recently been expressed in few published guidelines by US health organizations (namely, the US Department of Veterans Affairs, the American Heart Association, and the American Association of Clinical Endocrinologists). However, these recommendations have been worded at variable degrees of liberalization of lipid in the nonfasting state, with the US Department of Veterans Affairs being the most powerful recommendation [26].

### 4.1. Study Limitations

Our study has multiple limitations that would limit the findings and conclusions of the study. Furthermore, these limitations, as well, would prevent generalization of the findings to other populations. The first and foremost limitation of our study is that it was nonrandomized, and thus, the study findings will not comprise an accurate prevalence estimation. Therefore, this study is considered a prevalence study, but rather a study which observed the occurrence of FEEHD in a clinical setting. Secondly, our study was based on a survey dependent upon patients' recollection of hypoglycemic episodes. This may have led to an underestimation of prevalence, as patients with hypoglycemia unawareness may never have realized that their glucose is low. Another limitation is that we could not verify the exact reason for patients getting fasting tests. We rather have based our conclusions on our observation in our patients that lipid panels are the most commonly ordered fasting labs.

Given the aforementioned limitations, we acknowledge that this study could not be taken as an accurate estimate of the prevalence of FEEHD in the general population. It rather suggests that FEEHD is conceivably overlooked in clinical practice, and this second study by our group confirms the findings we reported in the previous, pilot study [16]. Therefore, we propose that larger, population-based studies be designed to evaluate the actual prevalence of FEEHD in the general population. Our group is working on such a project.

## 5. Conclusion

Despite the aforementioned limitations, our study does prove that there is a high rate of occurrence of iatrogenic fasting hypoglycemia resulting from laboratory tests in patients on medications which can induce hypoglycemia (FEEHD). Our study has confirmed findings of prior studies that FEEHD occurs in clinical practice and at an alarming prevalence rate. Ordering fasting lipid profiles will not only put patients with diabetes at risk of hypoglycemia, but with the changing guidelines in lipid testing, fasting for lipid tests may not be necessary after all. It is imperative that health organizations, especially diabetes organizations, become more aware of this issue and include specific educational guidelines to prevent FEEHD. As the utility of fasting lipid panel has been deemed doubtful and does not appear to be inferior to nonfasting measurements, it may now be high time to amend lipid management guidelines.

## Figures and Tables

**Table 1:** Characteristics of patients and a summary of survey responses.

Total number of patients invited to enroll in the study, *N*	572
Total number of patients who completed survey, *N* (survey response rate)	525 (91.8%)
Final analytic sample, excluding patients with no fasting labs (127) and patients with incomplete data (35)	363
Mean age (SD), years	60.6 (12.5)
Mean diabetes duration (SD), years	16.0 (11.5)
Sex, female	194 (53.2%)
Patients reporting type 2 diabetes mellitus, *N* (%)	298 (81.6%)
Patients on hypoglycemia-inducing OHAs^∗^ (without insulin)	36
Patients on insulin without hypoglycemia-inducing OHAs	215
Patients on both insulin and hypoglycemia-inducing OHAs	18
Total at-risk patients for FEEHD^∗∗^	269 (74.1%)
Total patients with FEEHD^∗∗∗^	62 (17.1%)
Patients with FEEHD from “at-risk” patient group (prevalence)	59 (21.9%)^∗∗∗∗^
Multiple FEEHD episode patients	7
Patients educated by health care prior to fasting labs	131 (35.9%)

FEEHD = fasting-evoked en route hypoglycemia in diabetes; SD = standard deviation; OHA = oral hypoglycemic agent. ^∗^Sulfonylureas and meglitinide analogues. ^∗∗^At-risk patients: patients who had fasting labs done and were on insulin or hypoglycemia-inducing OHAs or both. ^∗∗∗^FEEHD: fasting-evoked en route hypoglycemia in diabetes. ^∗∗∗∗^3 patients who had FEEHD were not on any hypoglycemia-inducing OHA or insulin. They were on metformin, saxagliptin, liraglutide, or canagliflozin.

## Data Availability

The data used to support the findings of this study are available from the corresponding author upon request.

## Abbreviations

FEEHD: Fasting-evoked en route hypoglycemia in diabetes.
